# How Does Organizational Unlearning Influence Product Innovation Performance? Moderating Effect of Environmental Dynamism

**DOI:** 10.3389/fpsyg.2022.840775

**Published:** 2022-08-10

**Authors:** Xiaoping Wang, Chenglin Zheng, Eugene Burgos Mutuc, Ning Su, Tianyu Hu, Haitao Zhou, Chuhong Fan, Feng Hu, Shaobin Wei

**Affiliations:** ^1^School of Public Affairs, Zhejiang University, Hangzhou, China; ^2^School of Economics and Management, Northeast Normal University, Changchun, China; ^3^College of Business Administration, Bulacan State University, Malolos, Philippines; ^4^School of MBA, Zhejiang Gongshang University, Hangzhou, China; ^5^School of Information Engineering, Zhengzhou University, Zhengzhou, China; ^6^Institute of International Business and Economics Innovation and Governance, Shanghai University of International Business and Economics, Shanghai, China; ^7^Institute of Spatial Planning & Design, Zhejiang University City College, Hangzhou, China

**Keywords:** organizational unlearning, dynamic capability, product innovation performance, environmental dynamism, knowledge management

## Abstract

Product innovation integrates technology, knowledge, management practices, and market innovation, making it essential to gain a competitive advantage. Effective management of dynamic knowledge, which is the foundation of and driving force for product innovation, is a powerful tool that allows a firm to successfully innovate, adapt to environmental changes, and improve its competitiveness. In the “nanosecond age,” unlearning and learning in an organization is crucial to a firm’s ability to promptly update its organizational knowledge and maintain innovation vitality. Based on the dynamic knowledge management perspective, this study integrates and constructs a theoretical model with environmental dynamism as the moderating variable, discusses the impact of organizational unlearning on product innovation performance, and empirically analyzes 208 valid questionnaires in the Yangtze River Delta using the multiple regression method. The results show that organizational unlearning shares a positive relationship with dynamic capabilities and product innovation performance. Dynamic capability is positively related to product innovation performance and has a partial mediating effect on the relationship between organizational unlearning and product innovation performance. Environmental dynamism shares a positive moderating effect on the relationship between organizational unlearning and product innovation performance. This study deepens the existing research on the factors that influence product innovation performance, which may help firms improve their dynamic knowledge management and product innovation performance.

## Introduction

Firms need to innovate, develop, and maintain competitive advantage in a dynamic and complex market environment (Nam et al., 2020). Among the different types of innovation activities, the most important and widely researched is product innovation, which is linked to firms’ profitability and long-term development (Cooper and Kleinschmidt, 1987; Zirger and Maidique, 1990; Cooper, 1991; Dias et al., 2020). Product innovation refers to how firms respond to changes in consumer demand and integrate and utilize resources to identify and develop products with better value propositions (Utterback and Abernathy, 1975; Calantone et al., 2003). It includes improvements to existing products as well as the innovation and development of new products (Levitt, 1966; Song and Montoya-Weiss, 1998). As China’s economy has entered a new stage of “structural adjustment, steady growth, and innovation-driven development,” product innovation provides an essential channel for firms to respond to technological and market changes and gain potential competitive advantages. Knowledge and innovation are closely related; knowledge is the foundation and driving force for innovation, and innovation is always supported by knowledge and information (Lichtenthaler, 2011). Therefore, studies emphasize the roles of new knowledge acquisition, perspectives, and methods as well as organizational learning and knowledge assimilation in product innovation (Brauner and Becker, 2006; Jensen et al., 2007; Bolaji and Adeoye, 2018). However, they ignore the management of invalid knowledge, which hinders organizational innovation. The accumulation of useless knowledge within the organization reduces the flexibility and agility of the organization, thereby becoming a stumbling block to enterprise innovation, and making the organization rigid and conservative (Leonard-Barton, 1992). In view of rapid changes in the environment, enterprises need to have certain flexibility and responsiveness, especially in the development of new products and technologies. Enterprises should “embrace changes” to deal with turbulent environments (Iansiti, 1995). Therefore, in the face of a changing environment, organizational personnel must have a variety of skills to deal with existing challenges, and one of the most important skills to doing so is “unlearning” (Nystrom and Starbuck, 1984; Erdogan and Tosun, 2009; Wang et al., 2013). Organizational unlearning is an organization’s ability to actively disrupt internal values, old ways of thinking, and outdated knowledge, helping firms innovate their thinking model, dominant logic, and cognitive structure (Cegarra-Navarro et al., 2016; Huang et al., 2018). Overcoming the rigidity of core competencies, which results from maintaining outdated conventions and concepts, is essential for promoting firms’ product innovation activities (Hamel and Prahalad, 1994; Sinkula, 2002). Scholars have begun to focus on the vital role of organizational unlearning in the product innovation process (Hedberg et al., 1981; Starbuck, 1996; Akgün et al., 2006); however, studies are scant. Compared to organizational learning, organizational unlearning is undervalued, which complicates the construction of a theory of unlearning.

Most managers and management scholars affirm the importance of organizational learning in improving firms’ product innovation performance, and substantial research supports this finding (Ramesh and Tiwana, 1999; Santos-Vijande et al., 2012). However, we cannot ignore the duality of organizational learning. Individuals and organizations cannot always learn blindly; it is necessary to abandon outdated ways of thinking, assumptions, behaviors, or conventions. If an organization cannot effectively unlearn or disconnect from its outdated and harmful tacit knowledge and entrenched cognitive structure, the organization’s cognitive inertia will limit its learning and absorption of new knowledge (Grisold et al., 2020) and inevitably restrict its innovative behavior. The key to an organization’s long-term development is balancing learning and unlearning (Gao and Zhu, 2020). Cultivating the ability to unlearn can prepare an organization to better interact externally, make timely responses to environmental changes (Hedberg et al., 1981), and prevent organizational rigidity and stagnation, all while creating room for innovation (Klammer et al., 2019). Nevertheless, the research findings on the impact of organizational unlearning on (product) innovation performance have always been controversial. These conclusions can be divided into three types. The first argues that organizational unlearning has a positive effect on (product) innovation performance in firms (Leal-Rodríguez et al., 2015; Huang et al., 2018). Second, organizational unlearning has a negative effect on (product) innovation performance in firms (Starbuck, 2017; Snihur, 2018). Finally, the third argument is that organizational unlearning does not have a direct effect on (product) innovation performance in firms (Siegal et al., 1996; Akgün et al., 2007). Scholars have studied the relationship between organizational unlearning and (product) innovation performance from different perspectives and, expectedly, have drawn different conclusions. In view of the differences between Chinese and Western enterprises in the macro political environment, meso industrial environment, and micro enterprise culture and values, most of the existing literature considers Western enterprises as the research object. Whether these conclusions are applicable to the Chinese situation needs to be further verified.

In addition, on the one hand, dynamic changes in the environment provide new opportunities for a firm to innovate; on the other hand, they weaken the adaptability of a firm’s core elements, including corporate values, culture, and knowledge structure. To better respond and adapt to changes in the external environment, the literature on dynamic capabilities argues that a firm must continuously improve its organizational agility and flexibility. Furthermore, dynamic flexibility (dynamic capability) is an important foundation for a firm’s success in innovation (Li et al., 2008). Thus, this study attempts to analyze the relationship between organizational unlearning, dynamic capabilities, and product innovation performance based on a combination of theories on organizational unlearning, knowledge, dynamic capabilities, and innovation. At the same time, this study examines the moderating effect of environmental dynamism within this relationship to enrich organizational management theories and empirical research, thereby providing strategic recommendations for managing unlearning and innovation in firms.

## Theoretical Analysis and Hypotheses

### Organizational Unlearning

Research on organizational unlearning was first conducted outside China, but most early foreign studies focused on passive unlearning. Organizational unlearning is defined from the perspective of the natural loss of knowledge in organizational circulation. It is considered as a process of attenuation, omission, and unconscious loss of organizational knowledge. That is, before the newly acquired knowledge and experience are transformed into organizational memory and entered into an organizational knowledge base, they will be affected by various factors (e.g., too many levels of knowledge transfer and deviation of understanding), leading to the unconscious unlearning of knowledge, or the degradation of old knowledge and old skills caused by changes in the organizational life cycle (Darr et al., 1995; Epple et al., 1996; Benkard, 2004). They also consider that its impact on organizations is often unfavorable. In their study of double-loop learning, Hedberg et al. (1981) state that “organizations will unconsciously unlearn the knowledge generated during the learning cycle,” which suggests that this kind of unlearning is passive. Othman and Hashim (2004) studied organizational learning processes and explained the reasons for and types of passive unlearning. Holan and Phillips (2004) argue that organizational unlearning is the opposite of organizational learning; that is, unlearning organizational knowledge impacts the execution of organizational tasks and, thus, negatively affects organizations. International scholars have gradually shifted their attention to active organizational unlearning and have argued that it is an important condition for organizations to successfully adapt to environmental changes. Subsequently, more studies began to examine organizational unlearning from the perspective of organizational learning and believed that the complete organizational learning process includes unlearning and not just learning (Hedberg et al., 1981). Organizational unlearning is considered an important supplementary form of organizational learning (Holan and Phillips, 2004) and an important prerequisite for the organization to carry out the relearning process (Nystrom and Starbuck, 1984). Hislop et al. (2014) argued that organizational unlearning is the process through which an organization loses memory (knowledge) to enable new learning. Rezazade Mehrizi and Lashkarbolouki (2016) find that organizational unlearning is a behavior of intentional forgetting, which aims to reduce an organization’s dependence on inherent old knowledge, conventions, and processes in favor of learning new knowledge. Baker and Sinkula (1999) also believe that when organizations actively question long-standing practices, assumptions, and beliefs, they engage in the practice of organizational unlearning. Furthermore, Yang et al. (2014) proposed that organizational learning includes forgetting old knowledge as well as changing beliefs and conventions within the organization. Finally, Sinkula (2002) and Akgün et al. (2003) argued that the essence of organizational unlearning is to change organizational beliefs and conventions. By contrast, Chinese research on organizational unlearning began relatively late, and, naturally, there has been little output. At present, research on organizational unlearning is mainly based on speculative and normative discussions that focus on analyzing the concept, connotation, and importance of organizational unlearning. The relevant empirical research is not only limited, but also has relatively low functionality, which has in turn affected the popularization and application of the theory of organizational unlearning.

Based on the above analysis, this study proposes that organizational unlearning is the process by which an organization actively abandons outdated intrinsic knowledge such as beliefs and conventions, which hinders organizational innovation and development. Furthermore, it lays the foundation for the organization to acquire new knowledge and build new beliefs and conventions. The organizational unlearning discussed in this study is an active and conscious unlearning behavior with four fundamental characteristics. The first is to “eliminate old,” sublating old knowledge such as outdated and useless beliefs and conventions in the organization. The second is to “accept, explore, and learn new knowledge to replace old knowledge.” The third is the “initiative.” Specifically, organizational unlearning reflects the organization’s will, and the organization actively and selectively discards solidified old knowledge in the organization. The fourth is “purposefulness.” That is, organizational unlearning is not only “elimination,” but also with more important purpose, namely, “renewal,” so as to prepare for the renewal of organizational beliefs and conventions, and the acquisition of new knowledge.

It is difficult for organizational knowledge systems to adapt to the needs arising from changes in the market, technology, and policy. Consequently, some knowledge may be outdated or develop into obstacles to the continued development of a firm. Amidst the same changes, the organization’s existing conventions, beliefs, and experiences may be inadvertently strengthened and become a competency trap, leading to inertia. Organizational inertia may also create barriers to sustainable innovation and reduce an organization’s responsiveness to the environment (Weidner et al., 2020). Specifically, scholars note that organizations may ignore critical new technologies and market changes because of their emotional investment in the widely established and accepted beliefs and practices with which they operate (Deiss, 1996). These established beliefs and practices create rules that harm operations (Mezias et al., 2001). For example, Day (1994) found that multiple successes rationalize past behaviors and practices, and the ensuing complacency may lead an organization to reject new information that conflicts with existing ideas. Thus, it becomes more difficult for an organization to learn, innovate products, or create if it cannot unlearn, which is why organizations must learn new conventions and forget old ones to adapt to environmental changes (Sinkula, 2002).

### Organizational Unlearning and Product Innovation Performance

The current dynamic business environment is characterized by competitive pressure and continuous improvement or development. As time passes, product life cycles become shorter, enabling firms to develop new products more quickly. Organizational knowledge is the firm’s leading source of innovation and development (Hu et al., 2021). The stronger a firm’s ability to update its knowledge, the more it can excel when competing to innovate, thereby enabling better innovation performance (Sanz-Valle et al., 2011). New product development and innovation require innovation teams to suspend existing concepts and methods (Starbuck, 1996). Innovation teams’ ability to adapt to a rapidly changing environment by unlearning old knowledge and learning the new facilitates transformation (Klein, 1989; Starbuck, 1996). Similarly, Tjosvold et al. (2004) took the new product development team as the object, and found that the new organizational beliefs and conventions formed by the organization through “unlearning-learning” can promote the development and innovation of new products. Through unlearning, new product development teams can create more innovative ideas and concepts in their minds; thus, unlearning before learning can drive successful innovation (Tsang and Zahra, 2008).

Unlearning is not simply a process of forgetting; it also involves renewing and replacing old conventions and ideas (Xi et al., 2020) or changing beliefs, norms, values, and procedures. These manifest as advancements in organizational concepts and conventions (Akgün et al., 2003). Unlearning behaviors are essential for product development activities at the organizational level (Buchen, 1999). Organizational unlearning can be used as a supplement to organizational learning to manage the organizational knowledge memory system effectively. The “cognitive inertia” and “core rigidity” of the organization can be reduced by removing obsolete concepts and conventions and other knowledge that are not relevant and timely (Cepeda-Carrión et al., 2012), after which the acute perception of new technical knowledge and demand changes of the organization can be improved (Akgün et al., 2007); therefore, the quality and utility of innovative products can be improved. Regardless of the extent of change, evolving concepts and conventions are a prerequisite for developing innovative products (Starbuck, 1996) because teams that experience success usually adopt similar concepts and conventions to develop new products. However, doing so will not produce pioneering ideas, nor will the team be able to integrate environmental changes (i.e., market and technology changes) into their development (Akgün et al., 2006), which in turn lowers the probability of launching innovative products in the future. Imai et al. (1988) note that Japanese firms have one advantage: they can flexibly adjust their strategies according to environmental changes during their product development process. According to Imai et al. (1988), unlearning can prevent a product development process from becoming rigid. Akgün et al. (2006) stated that project routines facilitate a fixed response to any information in a manner that does not require additional consideration. In addition, fixed concepts can lead to rigid perceptions or inaccurate causal attributions that slow down the speed at which organizations recognize change. The organizational memory theory states that organizations can practice unlearning to change or eliminate outdated and misleading knowledge and information. This practice facilitates the processing of new knowledge and enables organizations to act more flexibly under turbulent environmental conditions (Xi et al., 2021b). Therefore, organizations should selectively unlearn, update organizational memory, and optimize their knowledge systems to break away from inertia and rigidity to enhance their product innovation capabilities. Thus, we propose the following hypothesis:

Hypothesis 1: Organizational unlearning has a positive relation to product innovation performance.

### Mediating Effect of Dynamic Capabilities

Dynamic capability is a high-level comprehensive capability formed by enterprises based on organizational learning to perceive external opportunities, integrate and optimize the internal organizational resource base to create competitive advantages, and adapt to changes in the dynamic environment (Teece, 2007, 2014; Zahra et al., 2014). An ever-evolving external environment requires firms to monitor crises, flexibly identify opportunities and threats, and effectively coordinate and integrate internal and external resources and capabilities to improve the organization’s environmental adaptability. Dynamic capability is the core capability that a firm must possess to adapt to a changing environment and achieve sustainable development (Teece et al., 1997). What are the core elements that can improve an organization’s dynamic capabilities? Drucker (1993) once said, “In fact, knowledge is the only meaningful resource today. The traditional ‘factors of production’—land (i.e., natural resources), labor, and capital—have not disappeared, but they have become secondary.”

Throughout their life stages, organizations gradually accumulate operational knowledge, experience, standard operating procedures, conventions, beliefs, and culture. They store these elements of organizational knowledge in their memory systems to guide their practical activities. In a constantly changing environment, organizations find that their previous strategies, core capabilities (Prahalad and Hame, 1999), beliefs, values, and culture (Moorman and Miner, 1997) gradually decline in effectiveness and even become ineffective. These core competencies often require years of development and continuous improvement, but at the same time, they may become rigidities within organizations (Leonard-Barton, 1995) that hinder firms’ successes in the market. Organizational unlearning is not only the prerequisite and foundation for forming a firm’s dynamic capabilities but also drives their enhancements. Organizational unlearning can effectively facilitate the identification and elimination of outdated and inappropriate cognitive structures and conventions (Becker, 2008; Zhao et al., 2013), change the established thinking and working process to remove obstacles for learning and absorbing new knowledge from the outside, to keep the organizational knowledge base constantly updated and provide support for the organization to explore new innovations (Nonaka and Takeuchi, 1996; Lyytinen and Rose, 2003). Without unlearning, organizational memory systems cannot be updated, and dynamic capabilities cannot be enhanced. Casillas et al. (2010) found that as firms expand to international markets, they may unlearn irrelevant knowledge and conventions, which allows them to accelerate their exploration and new knowledge acquisition. Unlearning occurs when people need to update outdated knowledge structures. Moreover, unlearning may be a prerequisite for acquiring new knowledge, which means that it plays a vital role in forming and enhancing firms’ dynamic capabilities. Thus, we propose the following hypothesis:

Hypothesis 2: Organizational unlearning has a positive relation to dynamic capabilities.

Product development and innovation teams’ capabilities are often required to maintain competitiveness and develop strategic capabilities in a turbulent business environment. These dynamic capabilities refer to the perception, learning, integration, and coordination of an organization’s internal and external resources to respond to a rapidly changing environment (Pavlou and El Sawy, 2011). Dynamic capabilities are an essential element in enhancing a firm’s competitive advantage; they reflect an organization’s ability to gain a new competitive advantage based on its current market position (Leonard-Barton, 1992). Strong dynamic capabilities can help firms effectively build and update internal and external resources, and reallocate them as necessary to innovate and respond to or bring about changes in the broader market and business environment (Zahra et al., 2006; Teece, 2014).

Strategic management scholars indicate that when dynamic capabilities are aligned with corporate strategies, firms have a competitive advantage in new product development and innovation (Benedetto and Song, 2003; Harreld et al., 2007). Product innovation is a vital guarantee of firm success (Fain and Wagner, 2014). In essence, product development and innovation are knowledge-based activities that highlight the knowledge and learning processes in product development, production, and delivery. Dynamic capabilities can improve and enhance firms’ conventional capabilities (Prieto et al., 2009). This concept has been applied to research on innovation (Danneels, 2002; Huang et al., 2020), products, and process development (Benner and Tushman, 2003). Existing studies have revealed that dynamic capabilities can bring about positive results, including helping improve an organization’s competitive advantage (Eisenhardt and Martin, 2000; Wu, 2010), competitiveness (Marcus and Anderson, 2006), financial performance (Arthurs and Busenitz, 2006; Chen and Ma, 2021; Liu et al., 2021), and new product development performance (Park and Kim, 2013). Therefore, it is essential to strengthen the management of dynamic capabilities during product development and innovation to reap many performance-related benefits (Marsh and Stock, 2006). In their study of the hearing aid industry, Verona and Ravasi (2003) found that dynamic capabilities help companies develop and launch large numbers of high-quality products. Similarly, Park and Kim (2013), in their study of high-tech enterprises, found that dynamic capabilities can lead to better-performing new product development projects. Therefore, it can be said that dynamic capabilities are an important source through which firms can maintain high product innovation capabilities (Deeds et al., 2000). Thus, we propose the following hypothesis:

Hypothesis 3: Dynamic capabilities share a positive relationship with product innovation performance.

Unsuitable beliefs or thinking often leads to errors in judgment and actions; therefore, members of an organization may adapt their beliefs to change their perceptions of reality (Rousseau, 2001). This occurs because entrenched beliefs and conventions may make the organizational learning process path dependent. When a company receives positive feedback, its preceding beliefs and conventions are often reproduced, meaning that they may become the only beliefs and conventions that the company develops. This kind of rigidity further restricts firms from seeking new learning opportunities and performing their functions by acting upon them. Established conventions and beliefs may not only hinder organizations from searching for and adopting new ideas and knowledge, but they may also create regulatory, technical, and market-related misunderstandings, thus making it difficult for firms to adapt to turbulence in a changing environment (Ashforth and Fried, 1988).

Unlearning is a way to be freed from the learning inertia associated with the past environment (Hannan and Freeman, 1977). It is the organization’s strategic effort to liberate itself from knowledge that is no longer needed and to learn better and obtain more effective ways of doing so. Organizational unlearning is the process of replacing old patterns with new habits, beliefs, knowledge, and cognitive patterns (Cegarra-Navarro et al., 2010; Akhshik, 2014; Hislop et al., 2014), which can effectively promote organizational learning and the absorption of new knowledge, and creatively adjust existing products and technologies through the integrated application of new and old knowledge to create new products to adapt to the new environment (Hargadon and Sutton, 1997). Studies have found that in addition to reducing rigidity, organizational unlearning may promote the adoption of new knowledge and new technologies (Carlo et al., 2011), and reduce the interference of existing cognition and conventions in organizations’ pursuit of innovation and new developments (Shaft et al., 2008). In their study of the causes and consequences of unlearning in innovation teams, Akgün et al. (2006) proposed that by adjusting beliefs and conventions and integrating them into their project, team members may use this new knowledge and information to significantly improve the success rate of developing new products and help their firm cope better with fierce market competition. Firms can correct or delete outdated and incompatible knowledge and conventions within their organizations through unlearning (Wang et al., 2013; Zhao et al., 2013). They can also more flexibly explore and absorb new and external knowledge (Yildiz and Fey, 2010). Studies note that firms’ absorptive capacity can transform knowledge into new products, services, or processes that can support innovation (Cepeda-Carrión et al., 2012; Leal-Rodríguez et al., 2014) to cope with changes in the environment. Similarly, Sherwood (2000) emphasizes that the innovation process is achieved through two steps: (a) unlearning old and irrelevant knowledge and experiences and (b) collecting and using new innovation-related knowledge. Therefore, if an organization wants to develop innovative products, it should not only learn the latest technology and knowledge in time, but also give up outdated beliefs and conventions before that to obtain better product innovation performance. Thus, we propose the following hypothesis:

Hypothesis 4: Dynamic capabilities mediate the relationship between organizational unlearning and product innovation performance.

### Moderating Effect of Environmental Dynamism

Environmental dynamism describes the speed and unpredictability of changes in an external business environment. This is observed mainly from changeability in technological and market environments (Wang and Chen, 2010). The importance of environmental dynamism as a trigger for organizational unlearning has been confirmed in the organizational change and learning literature, and is most apparent in change models (Zhang and Zhu, 2021). Crisis resulting from environmental dynamism is one of the most important motivating factors for organizational unlearning. Organizational unlearning is achieved by adjusting perceptions and norms to adapt to learning needs in a changing environment and realizing a dynamic learning process (Wijnhoven, 2001). If the organizational knowledge base is compared to a reservoir with a valve, organizational learning constantly adds new knowledge to the knowledge base, which is similar to the process of water inflow, while organizational unlearning is the process of knowledge loss, similar to drainage. Due to limited space, the organizational knowledge base has an upper limit on the absorption and storage of knowledge. To learn and add new knowledge, an organization needs to first unlearn unnecessary or even harmful old knowledge to make room for the absorption of new knowledge (Cegarra-Navarro et al., 2012). Therefore, organizational knowledge forms a dynamic process based on “unlearning-learning” (Hedberg et al., 1981). Scholars note that organizations need to change their current beliefs, structures, norms, and conventions in a dynamically changing environment; otherwise, their existing beliefs and conventions may not be able to explain new realities when confronted with conflicting information (Starbuck, 1996; Sinkula, 2002). Cepeda-Carrión et al. (2012) proposed that organizations that want to innovate products or services must replace old knowledge. To respond better to environmental changes, firms must quickly adapt to them (Wallace et al., 2010; Zhou, 2021).

In the context of new product development and innovation, teams working in a dynamic environment encounter a rapid depreciation of technological and market knowledge due to changing customer needs, expectations, and technological proficiency. It is imperative to recognize this fact because team members’ knowledge will also become outdated and misleading, considering the rapid development of market and technological knowledge (Moorman and Miner, 1998; Xi et al., 2021a). In addition, researchers have found that, due to environmental changes, project plans, conventions, and procedures will become unproductive (Dickson, 1992) and may require organizations to evaluate and change their beliefs and methods to effectively address this new and conflicting information (Starbuck, 1996). Scholars state that unlearning activities, implemented in response to changes in beliefs and conventions (Sinkula, 2002; Akgün et al., 2006), can help organizations respond with greater flexibility to rapid changes in markets and technologies. Organizational unlearning may also accelerate the process of a firm’s evolution and adaptation to environmental changes, improve its competitiveness, and achieve innovation success. Cegarra-Navarro et al. (2016) and Love et al. (2018) proposed that environmental pressures in a dynamic environment have a more perceptible stimulating effect on organizational unlearning than in a stable environment. This makes it more conducive for firms to abandon outdated beliefs, conventions, and core rigidities and to promote innovation in firms. Thus, we propose the following hypothesis:

Hypothesis 5: Environmental dynamism has a positive moderating effect on the relationship between organizational unlearning and product innovation performance.

**FIGURE 1:**
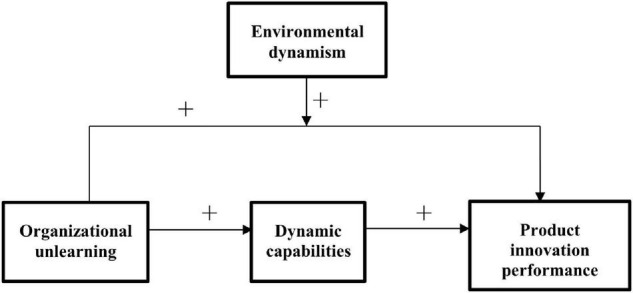
Theoretical model of the effect of organizational unlearning on product innovation performance.

Based on the above analysis, a relationship model that comprises organizational unlearning, dynamic capabilities, and product innovation performance in compliance with the “resource-capability-performance” logic is proposed (see Figure 1).

## Sample Selection and Data Collection

### Research Sample

This study considers a sample of manufacturing firms that were established for at least 3 years and engaged in product innovation activities. The questionnaire was mainly filled out by middle and senior managers who had worked in the sample firms for more than 3 years; they have a better understanding of the overall operation, product innovation, change process of organizational behavior and convention, and environmental challenges they face. Meanwhile, they also have a better understanding of the measurement items related to organizational unlearning, dynamic capabilities, product innovation performance, environmental dynamism, and other variables involved in the questionnaire to make the collected data more credible. After identifying the research subjects, the author distributed the research questionnaires across three channels. The first way is to teach MBA students at the author’s school. As MBA students are managers of enterprises, they are in line with the research requirements of this study. Following the recommendation of the MBA class teacher, the author invited MBA students to fill in the questionnaire on the spot. The second was to issue questionnaires through product fairs. The author searched for qualified enterprises by attending product fairs and invited middle and senior management personnel or technical personnel to fill in the questionnaires on-site. The third was to send questionnaires to the enterprise managers recommended by them in the form of emails through referrals from friends. In this study, 350 questionnaires were distributed, 305 were recalled, and 208 valid questionnaires were obtained after screening, with an effective rate of 68.2%.

The sample firms were mainly located in Hangzhou, Ningbo, Yiwu, Wenzhou, Taizhou, and Jiaxing in Zhejiang Province, China. Of these firms, 77.9% had been established for at least 5 years. In terms of firm size, 40.87% of the enterprises had more than 500 employees, and enterprises with fewer than 500 employees accounted for 59.13%. In terms of industry type, firms in the household electronics and communications, textile and clothing, and machinery and equipment manufacturing industries account for 15.4, 13.5, and 12% of the sample, respectively. Firms in the food/brewery/beverage/cigarette, transportation equipment, electronic equipment and communication equipment, computer and software, medical equipment, and chemical/plastic industries accounted for 11.0, 9.1, 8.7, 7.2, 6.7, and 6.3%, respectively. Few firms are in the consumer goods and furniture industries, accounting for 5.3 and 4.8% of the sample, respectively.

### Variable Measurement

This study applied a five-point Likert scale to analyze and measure all variables, except for the control variables. In this scale, the numbers from “1” to “5” express a respondent’s level of acceptance of a particular item, which gradually increases from “1” (strongly disagree) to “5” (strongly agree), and “3” (neutral) represents neither agree nor disagree.

The scale of product innovation performance mainly refers to Baker and Sinkula’s (1999) research results and consists of six items. Typical test items include: (a) “Our company improves the company’s profit through product innovation,” and (b) “Our company improves market share through product innovation.” The scale of organizational unlearning mainly draws on the research results of Akgün et al. (2007), which is measured from the two dimensions of belief change (six items) and convention change (six items). Typical test items include: (a) “with the development of the enterprise, the management style of our enterprise leaders (such as bold innovation, traditional conservatism, etc.) will change,” (b) “with the development of the enterprise, our enterprise’s concept of technology development trend will change,” and (c) “Our enterprise will introduce new knowledge that conflicts with previously recognized experience and skills.” The scale of dynamic capabilities was designed according to the research results of Teece (2007), Lin and Wu (2014), and Schilke (2014). It is measured using three dimensions: opportunity perception capability (six items), absorptive capacity (six items), and resource integration capability (four items). Typical test items include: (a) “Our enterprise often explores the needs of customers or potential customers,” (b) “Our enterprise often carries out cross-departmental learning activities,” and (c) “Our enterprise can successfully integrate new information obtained from the outside with known knowledge.” The scale of environmental dynamism mainly refers to the research results of Jaworski and Kohli (1993), and Wilden and Gudergan (2015), which were measured using the two dimensions of market dynamism (six items) and technological dynamism (three items). Typical test items include: (a) “In our business, customers’ product preferences change rapidly,” and (b) “In the industry in which our enterprise is located, technology changes very frequently.”

### Control Variables

This study finds that product innovation performance results from a combination of multiple factors. Studies have shown that a firm’s age, size, and industry type affect its innovation activities to a certain degree (Veugelers, 1997). Therefore, firm age, size, and industry type were included as control variables to reduce their effects on the research results and to highlight the effects of various variables in the proposed theoretical model of product innovation performance. Firm age was measured as the period since the firm was established, and firm size was measured using the current total number of employees, which is a common method adopted in existing studies (Deeds et al., 2000; Danneels, 2008). Firm size and firm age were then categorized into multiple groups based on studies conducted by Dibrell et al. (2011) and Li and Liu (2014).

## Data Analysis and Results

### Common Method Bias Test

Extensive sample data were tested for common method bias using Harman’s single-factor test. Accordingly, a factor analysis was conducted on all the measurement indicators included in the questionnaire, such as belief change and convention change in the organizational unlearning scale; opportunity perception capability, learning absorptive capability, and resource integration capability in the dynamic capabilities scale; and product innovation performance, market dynamism, and technological dynamism in the environmental dynamism scale. The test results showed that the eigenvalue of multiple factors was greater than 1, which explained 79.07% of the total variance. The highest explained variance among these factors was 17.18%, which was less than 20%, indicating that common method bias had little effect on this study.

### Reliability and Validity Tests

Cronbach’s α and the correction item total correlation (CITC) coefficient were used to test the reliability of the scales. The results show that Cronbach’s α values for the organizational unlearning, dynamic capabilities, product innovation performance, and environmental dynamism scales were all greater than 0.7. Additionally, the CITC values of all items were greater than 0.35. These results indicate that the scales used in this study have high reliability. For validity testing, confirmatory factor analysis (CFA) was conducted on the large-sample data based on a pre-test for small-sample data. The results show that the loadings of each variable measuring item were between 0.65 and 0.98, and most of them were greater than 0.70. The composite reliability (CR) of belief change and convention change in the organizational unlearning scale were 0.912 and 0.915, respectively, with average variance extracted (AVE) being 0.633 and 0.644 for each variable, respectively. The CR of opportunity perception capability, learning absorptive capability, and resource integration capability on the dynamic capabilities scale were 0.914, 0.947, and 0.852, respectively, with AVE values of 0.638, 0.750, and 0.592, respectively. The CR of the product innovation performance scale is 0.938, with an AVE of 0.718. On the environmental dynamism scale, market dynamism and technological dynamism showed a CR of 0.978 and 0.979, respectively, and AVE of 0.883 and 0.940, respectively. All values were greater than 0.5, indicating that the scales used in this study have good construct validity and good overall model fit.

### Analysis of Descriptive Statistics and Correlation Coefficients

Table 1 presents the mean, standard deviation, and correlation coefficients for each variable. The results show that organizational unlearning was significantly correlated with dynamic capabilities (*r* = 0.770, *p* < 0.01), product innovation performance (*r* = 0.799, *p* < 0.01), and environmental dynamism (*r* = 0.194, *p* < 0.05). Moreover, product innovation performance is significantly correlated with dynamic capabilities (*r* = 0.794, *p* < 0.01) and environmental dynamism (*r* = 0.211, *p* < 0.05). These results indicate that the key variables in this study are correlated, and that the model has scientific rationality.

**TABLE 1:** Mean, standard deviation, and correlation coefficients of variables.

Variable	1	2	3	4	5	6	7
1 Firm age	1						
2 Firm size	0.381**	1					
3 Industry type	−0.231**	−0.210**	1				
4 Organizational unlearning	0.118	–0.003	–0.142	1			
5 Dynamic capabilities	0.102	0.052	–0.154	0.770**	1		
6 Product innovation performance	0.101	–0.049	–0.132	0.799**	0.794**	1	
7 Environmental dynamism	0.113	–0.061	–0.041	0.194*	0.133*	0.211*	1
Mean	3.830	2.980	6.060	3.793	3.874	3.696	3.267
Standard deviation	1.190	1.683	2.801	0.552	0.619	0.689	1.252

*p < 0.05, **p < 0.01.

### Main Effect Test

Hierarchical regression analysis was employed in this study, and the results are shown in the M2 section in Table 2. After controlling for firm age, size, and industry type, the result of organizational unlearning (regression coefficient) on product innovation performance was 0.673 (*p* < 0.001), indicating that organizational unlearning has a significant positive relationship with product innovation performance. Therefore, Hypothesis 1 was supported.

**TABLE 2:** Results of hierarchical regression analysis (*N* = 208).

Variable	Product innovation performance					Dynamic capabilities		
	M1	M2	M3	M4	M5	M6	M7	M8
Firm age	0.117	0.039	0.033	0.053	0.002	0.028	0.103	0.021
Firm size	–0.120	–0.070	–0.096	–0.148	–0.055	–0.054	0.044	0.097
Industry type	–0.130	–0.042	–0.016	–0.011	–0.040	–0.026	−0.191*	–0.098
Dynamic capabilities			0.264**	0.620***				
Environmental dynamism					0.181**	0.210**		
Organizational unlearning		0.673***	0.486***		0.652***	0.601***		0.708***
Organizational unlearning × environmental dynamism						0.226***		
F	1.549	28.747***	25.877***	20.845***	25.816***	25.831***	3.006*	39.076***
R^2^	0.035	0.473	0.505	0.394	0.504	0.552	0.065	0.550
Adjusted R^2^	0.012	0.457	0.485	0.376	0.485	0.530	0.044	0.546
△R^2^		0.438	0.032	0.359	0.469	0.048		0.485
D-W	2.104	1.941	1.951	2.092	1.995	2.064	1.862	1.993
VIF_max_	1.203	1.217	2.221	1.214	1.259	1.273	1.203	1.217

*p < 0.05, **p < 0.01, and ***p < 0.001.

*The values listed in the table are standardized regression coefficients.*

### Mediating Effect Test

The mediating effect test recommended by Baron and Kenny’s (1986) study was employed, and the results are as follows. First, as shown in M2, organizational unlearning has a significantly positive relationship with product innovation performance (β = 0.673, *p* < 0.001). Second, as shown in M8, organizational unlearning has a significantly positive relationship with dynamic capabilities (β = 0.708, *p* < 0.001). Thus, Hypothesis 2 was supported. Third, as M4 shows, dynamic capabilities have a significantly positive relationship with product innovation performance (β = 0.620, *p* < 0.001). Thus, Hypothesis 3 is supported. Fourth, comparing M3 and M2, the effect of organizational unlearning on product innovation decreases from 0.673 to 0.486 in M2 and M3, respectively, but remains significant after introducing dynamic capabilities. This indicates that dynamic capabilities partially mediate the relationship between organizational unlearning and product innovation performance. Thus, Hypothesis 4 is supported.

### Moderating Effect Test

As shown by M6 in Table 2, the interactive term between organizational unlearning and environmental dynamism has a significantly positive effect on product innovation performance (β = 0.226, *p* < 0.001). This result shows that environmental dynamism has a positive moderating effect on the effect of organizational unlearning on product innovation performance. Thus, Hypothesis 5 is supported.

## Main Research Conclusion and Contributions

### Research Conclusions

Based on theories of organizational unlearning, learning, knowledge management, innovation, and dynamic capabilities, this study constructs a mechanistic model of how organizational unlearning affects product innovation performance from a dynamic capabilities perspective. Empirical testing of 208 valid samples revealed several findings. First, organizational unlearning has a significantly positive relationship with product innovation performance. This conclusion is consistent with the findings of Akgün et al. (2007), Tsang and Zahra (2008), and Becker (2010). By unlearning behavior and abandoning internal solidified old knowledge such as cognitive ideas, concepts, practices, and behavior norms, organizations can not only eliminate the excessive dependence on previous successful models, but also make room for absorbing new knowledge, so as to generate opportunities to seek new ideas and promote the improvement of enterprise product innovation performance (Zahra et al., 2011). Second, organizational unlearning has a significantly positive relationship with dynamic capability. In the context of organizational strategies, unlearning occurs actively. As organizations grow and become more complex, in order to maintain their flexibility in the dynamic environment, they must strive to break the rules that guided their success in the past; that is, they need to change and adjust their ideas, procedures, systems, practices, and processes to comply with the market-oriented logic that interacts with them (Bettis and Prahalad, 1995), and improve their ability to perceive external opportunities and practice opportunities through innovation. Third, dynamic capabilities are important internal capabilities that affect product innovation performance, and organizational unlearning can promote product innovation performance through dynamic capabilities. The core of unlearning behavior lies in trying to reposition organizational values, norms, practices, and behaviors to gain a competitive advantage by changing the organizational cognitive structure (Nystrom and Starbuck, 1984), psychological model (Day and Nedungadi, 1994), and dominant logic and core assumptions guiding the behavior (Bettis and Prahalad, 1995). Organizational unlearning can create conditions for shaping enterprises’ dynamic capabilities and product innovation. By unlearning behavior, organizations can promote the “dynamicity” of their capabilities, learn to absorb new knowledge, perceive and take advantage of new opportunities, create new asset portfolios, and innovate products to meet new market demands (Cepeda and Vera, 2007), so as to improve the product innovation performance of enterprises. Four, environmental dynamicity positively moderates the effect of organizational unlearning on product innovation performance. This conclusion is similar to that of Cegarra-Navarro et al. (2016). In a dynamic environment, the effect of organizational unlearning is better. Because changes in the environment will trigger organizational unlearning, enterprises can actively engage in unlearning behavior under external pressure, abandon invalid knowledge, learn and absorb new knowledge, and establish brand-new organizational concepts and practices to help enterprises eliminate capacity rigidity, so as to promote the innovation and R&D of new products, meet competitive demands, and keep pace with the development of the times through product innovation (Akgün et al., 2007). In summary, the hypotheses proposed in this study were supported. Based on the research conclusions, this study emphasizes that enterprises need to pay attention to organizational unlearning based on organizational learning. By constructing an “unlearning-learning organization,” the dynamic management of organizational knowledge can help enterprises eliminate core rigidity, enhance innovation vitality, and thus promote the improvement of product innovation performance.

### Theoretical Contributions

Through theoretical discussion and empirical testing, this study constructs and verifies the mechanism that analyzes the effects of organizational unlearning on product innovation performance and reveals the inner workings of this effect, thereby making several contributions to research in related fields.

First, this study introduces the unlearning theory based on reverse thinking to examine the effect of organizational unlearning on product innovation performance from the perspective of knowledge “subtraction.” This verifies that organizational unlearning has a significantly positive relationship with product innovation performance. It also confirms that organizational unlearning enhances product innovation performance through dynamic capabilities. This conclusion reveals, to a certain extent, the internal mechanism through which organizational unlearning affects product innovation performance. It not only expands and enriches research on the antecedents of product innovation performance but also provides a new perspective for research on knowledge management.

Second, studies propose that organizational unlearning has a positive effect on firms’ innovation (Tsang and Zahra, 2008), while others argue that organizational unlearning has a negative impact on innovation because it may consume limited organizational resources and cause confusion or fear among organizational members (Akgün et al., 2007). This study introduces dynamic capabilities as a mediating variable and finds that organizational unlearning has direct and indirect effects on product innovation performance. This conclusion not only validates the “resource-capability-performance” logic, but also enriches organizational unlearning and innovation theories. Therefore, this study serves as a valuable reference for resolving disputes in the mainstream literature.

Finally, the fact that previous studies overlooked control variables may have affected their research conclusions. By controlling for the age, size, and industry type of the Chinese manufacturing firms in the sample, this study verified that organizational unlearning has a positive relationship with product innovation performance. This conclusion is consistent with the research conclusions of Leal-Rodríguez et al. (2015) and Huang et al. (2018), which further support the first type of opinion in the academic debate. In addition, this study verified the positive moderating effect of environmental dynamism on the effect of organizational unlearning and product innovation performance. This conclusion expands on and enriches the research on the situational factors that exist in the relationship between organizational unlearning and product innovation performance, which is of great significance.

### Managerial Implications

The conclusions drawn in this study are of great significance in guiding innovation management in Chinese firms. Therefore, this study proposes the following constructive management recommendations for firms.

First, managers should foster a culture of organizational unlearning to build an “unlearning-learning organization.” Harvard Professor Levitt (1966) stated that the biggest advantage of a successful organization is that it can quickly and purposefully outgrow past successes and achievements. Therefore, managers should strategize organizational unlearning and institutionalize it to build an “unlearning-learning organization.” An “unlearning-learning organization” is a higher-level organization type, which requires organizations to take the initiative to evaluate and screen existing internal knowledge and promptly discard outdated and invalid knowledge to promote the organizational learning process. By doing so, organizations can realize the dynamic cycle of “reviewing-evaluating-negating-discarding-relearning” organizational knowledge, which allows them to eliminate the rigidity of their core capabilities and establish a foundation for enhancing organizational creativity. Enterprise managers should actively guide the thinking mode of employees to keep pace with the times, encourage them to emancipate their minds, constantly examine the beliefs and conventions within the organization with an open attitude, help them get rid of the shackles of the thinking set, shape new ideas by absorbing new knowledge, and then improve their innovation ability.

Second, managers should establish a management mechanism for proactive organizational unlearning. Managers should actively improve their organization’s “unlearning and learning” mechanism by formulating policies that incentivize organizational unlearning, encouraging members to change their mindsets, proactively abandoning outdated and obsolete beliefs and conventions, and accepting new insights and knowledge with an open and innovative attitude. Moreover, managers should actively guide and control the direction and process of updating organizational beliefs and conventions. Managers should interactively update their firms’ pathway toward sustainable development by comprehensively considering changes in the external environment and the current developmental status of their firms. It should be noted that organizational unlearning cannot be achieved overnight; it must be implemented gradually and methodically. Hasty implementation is likely to cause psychological rejection by organizational members. Based on this, first of all, managers should create a relaxed, friendly and trustable “people-oriented” corporate culture for the organization, and create a good atmosphere for the organizational unlearning. Secondly, managers should use incentive strategies to guide employees to develop the good habit of “unlearning-learning.” Finally, managers should use material rewards to affirm employees’ unlearning and innovation behaviors.

Finally, managers should pay attention to changes in the external environment and make firm capabilities more dynamic by updating organizational knowledge and memory. Knowledge forms the basis of these capabilities. The existing knowledge of an organization is based on its previous environment. When there is a change in the external environment, knowledge in the organization’s knowledge base will no longer be applicable. Therefore, firms must continuously update their organizational knowledge and memory database when facing a dynamic and changing external environment, and know when to reset them. Firms should discard useless knowledge and simultaneously absorb new knowledge to update their knowledge structures, thereby transforming static organizational knowledge into dynamic capabilities through dynamic knowledge management. In doing so, firms’ capabilities become dynamic. Consequently, firms can break away from the rigidity of capabilities and path dependence in the innovation process and promote the organized development of product innovation activities.

### Research Limitations and Future Prospects

Owing to limitations in research capabilities, resources, and other conditions, this study has limitations that may be overcome in subsequent studies. First, this study used cross-sectional data for analysis. However, it takes time for firms to update their organizational beliefs, change conventions, and expand their dynamic capabilities to produce better results. Moreover, organizational unlearning is a gradual and dynamic process, whose effect on product innovation performance too is gradual, long-term, and dynamic. Therefore, using only cross-sectional data to study internal mechanisms will inevitably have shortcomings. Therefore, future studies should consider conducting a longitudinal design to analyze the process through which dynamic changes in organizational beliefs and conventions and the evolutionary development of dynamic capabilities affect product innovation performance. Furthermore, they may also integrate the firm life cycle theory to explore whether the effects of organizational unlearning and dynamic capabilities on product innovation performance differ at different stages of a firm’s life cycle. Second, studies on product innovation performance show that industry type affects product innovation performance in different ways. This study only considers manufacturing enterprises as the research object in general and does not distinguish between specific industries; therefore, the pertinence of the research conclusions to other industries may not be strong. Future studies may perform a comparative analysis of whether the effect of organizational unlearning on product innovation performance varies by industry type, especially between high-tech and traditional manufacturing industries, to provide relevant recommendations for management in different industries. They may also focus on firms within a single industry. Third, the sample firms in this study are mainly based in the Yangtze River Delta region, which is a relatively small region; therefore, the universality of the research conclusions may be relatively low. Whether the research conclusions apply to firms in other regions of China remains to be determined. Therefore, future studies can consider expanding the geographic scope of samples by including firms in China’s representative economically developed regions, such as the Pearl River Delta region, the Beijing-Tianjin-Hebei region, and the Yangtze River Delta region. Expanding the scope of the sample would increase the universality of the conclusions. Future studies may also consider collecting information on sample firms from different regions to conduct a cross-regional comparative study and provide managerial implications and recommendations for firms in different regions.

## Data Availability Statement

The original contributions presented in this study are included in the article/supplementary material, further inquiries can be directed to the corresponding author.

## Author Contributions

XW performed the research, writing, data collection, and analysis of the manuscript. FH provided the research theme and designed the research framework, while other authors provided help with data collection and translation of the study. All authors have read and agreed to the published version of the manuscript.

## Conflict of Interest

The authors declare that the research was conducted in the absence of any commercial or financial relationships that could be construed as a potential conflict of interest.

## Publisher’s Note

All claims expressed in this article are solely those of the authors and do not necessarily represent those of their affiliated organizations, or those of the publisher, the editors and the reviewers. Any product that may be evaluated in this article, or claim that may be made by its manufacturer, is not guaranteed or endorsed by the publisher.
